# COVID-19 Excess Deaths in Peru’s 25 States in 2020: Nationwide Trends, Confounding Factors, and Correlations With the Extent of Ivermectin Treatment by State

**DOI:** 10.7759/cureus.43168

**Published:** 2023-08-08

**Authors:** Juan J Chamie, Jennifer A Hibberd, David E Scheim

**Affiliations:** 1 Data Analysis, Independent Data Analyst, Cambridge, USA; 2 Faculty of Dentistry, University of Toronto, Toronto, CAN; 3 Commissioned Corps, Inactive Reserve, United States Public Health Service, Blacksburg, USA

**Keywords:** excess deaths, ivermectin, peru, sars-cov-2, covid-19

## Abstract

Introduction

In 2020, nations hastened to contain an emerging COVID-19 pandemic by deploying diverse public health approaches, but conclusive appraisals of the efficacy of these approaches are elusive in most cases. One of the medicines deployed, ivermectin (IVM), a macrocyclic lactone having biochemical activity against SARS-CoV-2 through competitive binding to its spike protein, has yielded mixed results in randomized clinical trials (RCTs) for COVID-19 treatments. In Peru, an opportunity to track the efficacy of IVM with a close consideration of confounding factors was provided through data for excess deaths as correlated with IVM use in 2020, under semi-autonomous policies in its 25 states.

Methods

To evaluate possible IVM treatment effects, excess deaths as determined from Peruvian national health data were analyzed by state for ages ≥60 in Peru’s 25 states. These data were compared with monthly summary data for excess deaths in Peru for the period 2020-2021 as published by the WHO in 2022. To identify potential confounding factors, Google mobility data, population densities, SARS-CoV-2 genetic variations, and seropositivity rates were also examined.

Results

Reductions in excess deaths over a period of 30 days after peak deaths averaged 74% in the 10 states with the most intensive IVM use. As determined across all 25 states, these reductions in excess deaths correlated closely with the extent of IVM use (p<0.002). During four months of IVM use in 2020, before a new president of Peru restricted its use, there was a 14-fold reduction in nationwide excess deaths and then a 13-fold increase in the two months following the restriction of IVM use. Notably, these trends in nationwide excess deaths align with WHO summary data for the same period in Peru.

Conclusions

The natural experiment that was put into motion with the authorization of IVM use for COVID-19 in Peru in May 2020, as analyzed using data on excess deaths by locality and by state from Peruvian national health sources, resulted in strong evidence for the drug's effectiveness. Several potential confounding factors, including effects of a social isolation mandate imposed in May 2020, variations in the genetic makeup of the SARS-CoV-2 virus, and differences in seropositivity rates and population densities across the 25 states, were considered but did not appear to have significantly influenced these outcomes.

## Introduction

The COVID-19 pandemic swept through Peru in early 2020, with its first identified case recorded on February 26, 2020 [1]. Prior to mandating the use of COVID-19 vaccines, Peru relied upon the mitigation strategies of lockdowns and the introduction of therapeutics, as did other nations. Peru’s national lockdown began on May 16, 2020 and was extended through the end of June [2]. As one therapeutic option, on May 8, 2020, the Peruvian Ministry of Health approved treatment using ivermectin (IVM) [3], a drug of Nobel prize-honored distinction that has been used in 3.7 billion human doses worldwide since 1987 [4-6]. Within months, as reviewed below, results of randomized clinical trials (RCTs) for IVM treatment of COVID-19 began to be published.

Following the authorization on May 8, 2020, each of the 25 states of Peru implemented inpatient and outpatient treatments with IVM to different extents and in different time frames, as detailed below. The government of Peru independently tracked two indices of the pandemic’s mortality, state by state, daily: COVID-19 case fatalities and all-cause deaths, the latter index enabling calculations of excess deaths. Further complicating the epidemiological record, on November 17, 2020, a new president of Peru, Francisco Sagasti, took office [7]. Government distributions of IVM, the channel by which most patients had obtained it previously, were then stopped, with its further use allowed only by a doctor’s prescription [8-12]. Nationwide changes in daily excess all-cause deaths before and after these restrictions in IVM use will be presented in the Results section.

As detailed in the section on data sources, the data analyses presented here were performed in 2021, with the underlying Peruvian health dataset snapshot taken on December 13, 2020 used for the main analysis and another health dataset snapshot taken on February 23, 2021 corresponding to an analysis of changes in nationwide excess deaths both deposited and freely available in the Dryad data repository [13,14]. Notably, the February 23, 2021 data snapshot closely aligned with the most recently available Peruvian national health data and with WHO summary data for excess deaths in Peru, in the period 2020-2021, with the comparison procedures as detailed [14].

Conclusive assessments of the effects of drug distributions and treatments on a national scale, however, are typically precluded by confounding influences. One such potential pitfall with the analysis of population-level mortality data is the use of case fatality statistics, which are often unreliable and which were indeed underreported for COVID-19 in Peru [15]. In addition, the unstratified aggregation of all age groups could distort this analysis. Different states in Peru have varying age distributions, and the percentages of COVID-19 cases across age groups could change through the course of the pandemic. Another extraneous influence that could have affected COVID-19 mortality in Peru was a diversity of genetic lineages of SARS‑CoV‑2 that circulated at various incidence rates state by state in Peru and worldwide in 2020 [16,17]. Moreover, a social isolation mandate imposed nationally in May 2020 would have affected death rates from COVID-19 in Peru. Other confounding factors were varying seropositivity rates and population densities in the different Peruvian states, as detailed below in the Discussion section.

The availability of robust data for these potentially confounding influences and for excess death values by age allowed an assessment of these distortions and the derivation of reliable conclusions. Moreover, facilitating analysis of correlations between IVM usage and excess deaths were robust variations in the extent and timing of IVM distributions in the 25 Peruvian states. In 10 of these states, IVM was distributed widely within brief periods that began at different times. Close analysis was thus possible to check for correlations of excess deaths with the extent and timing of IVM distributions in Peru’s 25 states. In particular, for the 10 states with mass IVM distributions, changes in excess deaths could be compared with the start dates of the IVM distributions. To minimize potential distortions caused by varying proportions of younger or older people in the different states or in their shifting pools of COVID-19 patients, all analyses except one for changes in nationwide excess deaths, which was presented to indicate overall trends rather than to draw statistical inferences, were restricted to the population age ≥60. Since 75% of the excess deaths linked to COVID-19 during the pandemic’s first wave in Peru in 2020 were in the subgroup of age ≥60 [18], this restriction did not significantly change the analysis, but it eliminated one potential confounding element. The following background on the 25 geopolitical entities of Peru and on IVM and its use in Peru in 2020 sets the stage for consideration of the study design and analytical methodology.

The 25 states of Peru

Peru is divided into 24 *departamentos*, one of these being the Lima capital region, plus the independent *provincia* of Callao, which lies entirely within Lima [19]. For simplicity of reference, these are designated here as the 25 states of Peru. Mass distributions of IVM for the inpatient and outpatient treatments of COVID-19 occurred autonomously in these 25 states through both public and private channels. IVM treatments began in different time periods between April and August 2020 in each of the 25 Peruvian states. In some, they began even a few weeks before the May 8 national authorization. Details of IVM distributions from these public and private sources in nine representative states, spanning different latitudes and terrains, have been provided [20]. The 25 states of Peru, with a combined total population of 33 million, span terrains from jungles to deserts to mountains, equivalent to an extent from Denmark to Italy and Greece in Europe or from Florida to Minnesota to New York in the United States.

Distributions of IVM in the 25 states of Peru in 2020

A description of IVM distributions and treatments in Peru with state-by-state values for excess deaths and COVID-19 case fatalities was presented in a previous analysis [20]. In each state of Peru except Lima, IVM treatments were widely deployed at the time of the initial surge of pandemic cases and deaths; that surge period varied among the 25 states between April and August 2020. The typical dose of IVM provided to both COVID-19 inpatients and outpatients was 200 µg/kg for a single day for mild cases, repeated a second day for more serious cases [3].

Public compliance with these IVM treatments was achieved with well-publicized reports of successful outcomes for the IVM treatments of COVID-19 by Peruvian celebrities [20]. The level of popular interest in IVM treatments for COVID-19, as spurred by these reports, was so high that it led to IVM shortages in Peruvian pharmacies [21], which motivated smugglers [22] and counterfeiters [23] to cover the demand. In the Lima capital region, however, restrictive measures on IVM distribution, including police raids on pharmacies, delayed mass IVM treatments for COVID-19 for four months after the initial pandemic surge in April [20,24,25]. Finally, in August 2020, after 10,386 COVID-19 case fatalities had been recorded in Lima, 1.0 per thousand total population, through July 31 [26], IVM distributions and treatments began there on a large scale [20].

*Mega-Operación Tayta* (*MOT*)

IVM was typically distributed through regional health offices, voluntary channels, and other private groups, as detailed for several states [20]. However, 10 states distributed IVM on a mass scale through a national program led by the Ministry of Defense, *Mega-Operación Tayta* (*MOT*). Two of these states had confounding factors for their distributions of IVM. Pasco had three different distribution dates, July 23, August 5, and August 25 [27-29], while Junin’s *MOT* deployment, which began August 4, had been preceded by state distributions of IVM to health centers beginning July 22 [30,31], 13 days earlier.

*MOT*, an extension of a precursor program, *Operación Tayta* [32], was spearheaded by the Peruvian Ministry of Defense and Army. Eleven other government agencies partnered in this effort, including the ministries of health, interior, agriculture, and education, while participating personnel included those from the Army, Navy, Air Force, and police [33]. *MOT*’s objective was to reach every part of a targeted region using rapid response teams that partnered with local health officials. These teams detected COVID-19 cases house by house, administered IVM to patients and family members in their households, and gave them food to encourage their isolation for 15 days [34].

In each targeted locality, operation *MOT* began with outreaches, including home visits, by local officials to identify people at highest risk for COVID-19 mortality, due to either age or other vulnerabilities [35]. No IVM was distributed through *MOT* during this preparatory period, but it was freely available everywhere in Peru without a prescription, and people identified as vulnerable had the capability to purchase and take it during that time on their own initiative. A week later, field workers from *MOT* then began the distribution of IVM to everyone identified as being at risk, whether or not they tested positive or were symptomatic for COVID-19 [35]. Other drugs commonly distributed along with IVM were acetaminophen and azithromycin [10,36]. *MOT* began in late July 2020 and reached these 10 states, with *MOT* start dates as specified, designating the beginning of the preparatory week: Cajamarca (July 23) [37], Pasco (July 23, August 5 and August 25) [27-29], Moquegua (July 30) [38,39], Junín (August 4) [40], Puno (August 7) [41,42], Huánuco (August 7) [36,43], Huancavelica (August 7) [44], Ayacucho (August 13) [45], Cusco (August 13) [45], and Tacna (August 14) [46].

Background on IVM treatment of COVID-19

Since May 8, 2020, when IVM was authorized for COVID-19 treatments in Peru, inpatient and outpatient treatments of COVID-19 with IVM have been deployed in 25 countries [4]. More than 20 RCTs have been conducted for such IVM treatments, most of which indicated efficacy for IVM, as reviewed [4,47,48]. By contrast, some prominently cited RCTs for IVM treatment of COVID-19 performed in 2021 [49,50] and 2022 [51,52] had negative outcomes, but some of these RCTs had major flaws, which called their findings into question. One of these switched IVM and placebo doses for 38 patients, systematically violated blinding, and showed distinctive signs of IVM use in the placebo group [49,53]. In another of these RCTs, those distinctive adverse effects for IVM (transient and not serious), which typically show up at 20-30% incidences, including nausea and dizziness, were reported at nearly identical rates of less than 1% in both the treatment and placebo groups, and the study did not specify its source of IVM [52,54].

Coauthors of the 2022 TOGETHER Trial, which reported a negative conclusion for IVM treatment of COVID-19 [51], repeatedly refused to disclose four per-protocol outcomes of paramount interest, two each for deaths and hospitalizations [55]; both the National Institute of Health (NIH) and Food and Drug Administration (FDA) deemed the platform trial’s primary outcome as inadequate [56,57]. One of the TOGETHER Trial coauthors directed investigators to a data repository that never held the study’s data [55]; he also coauthored three subsequent RCTs that likewise concluded that IVM lacked treatment efficacy against COVID-19 [58-60]. An August 2022* New England Journal of Medicine* (NEJM) editorial categorically dismissed IVM as ineffective [61], citing as a centerpiece of its case a June 2022 meta-analysis of IVM treatment studies for COVID-19 [62]. Remarkably, however, in contradiction to that editorial’s conclusion, the first primary outcome that the meta-analysis reported was an odds ratio of 0.51 for IVM versus placebo for mortality, which amounted to a two-fold reduction in deaths.

Among the subjects of current research related to the evaluation of IVM efficacy against COVID-19 is the underlying biochemistry, notably competitive binding by IVM to SARS-CoV-2 spike protein attachment sites that appear to mediate the virus’s morbidity [63-65]. This molecular mechanism could explain the sharp increases in SpO_2_ (peripheral oxygen saturation) values observed in severe COVID-19 patients within 24 hours after IVM treatments in three studies, which tracked SpO_2_ values without the use of supplemental oxygen [66-68].

IVM is practical for widespread distribution on a global scale, having been deployed in worldwide campaigns to eradicate two scourges, onchocerciasis and lymphatic filariasis [69]. It holds a 34-year record of safety [4,6,70], including at doses considerably higher than the standard dose of 200 μg/kg [71-73]. It was used in RCTs for COVID-19 treatment at cumulative doses of 1,500 μg/kg [49], 3,000 μg/kg [74], and 6,000 μg/kg [75] over five days with only small percentages of mild or transient adverse effects.

This article was published as a reprint on OSFPrints in 2021 [106]. The analysis presented here extends and refines the work reported in the preprint by the same authors.

## Materials and methods

Study design

An ecological study design was used. The health tracking values used for the analysis were compiled daily by the *Centro Nacional de Epidemiología, Prevención y Control de Enfermedades* (National Center for Epidemiology, Prevention and Disease Control) and *Instituto Nacional de Salud* (National Institute of Health, NIH) in Peru. The tracking value used for the analysis was deaths from all natural causes (excluding violent deaths), hereinafter termed “all-cause deaths.” As discussed in a prior comprehensive analysis of IVM distributions and mortality trends in Peru [20], case incidence is an unreliable statistic across a national population and was not considered here. Case fatality statistics for COVID-19 in Peru [14], which were found to have been underreported [15], were also not used in the analysis.

Data sources

The data source for all-cause deaths used in this analysis was the registry of Peru's National Information System of Deaths (SINADEF) [76], with associated frozen datasets available from the Dryad data repository [13]. This SINADEF database for all-cause deaths was structured to record each death with one database record containing fields for age, sex, locality, and several other demographic characteristics. Data for populations, by state and by age groups, are from Peru’s National Institute of Statistics and Informatics [77]. Data for COVID-19 case fatalities, which as noted were referenced only peripherally, were from the Peruvian government’s COVID-19 Open Data Platform [26]. The databases for both all-cause deaths and COVID-19 case fatalities are subject to occasional retroactive adjustments, for example, if a death in a remote location was initially reported days after it occurred. These occasional adjustments have very small impacts on aggregate statistics, but database access dates are cited and frozen database snapshots were saved for all values presented. Further details on the contents of these Peruvian national health databases and on cross-checking performed with more recent data snapshots and with WHO monthly summary data for excess deaths in Peru, 2020-2021, are as described [14]. Data for IVM distributions were retrieved from official communications and press releases, as individually cited, and from the *Centro Nacional de Abastecimiento de Recursos Estratégicos en Salud *(CENARES, National Center for the Supply of Strategic Health Resources) drug distribution database [20].

Determinations of excess deaths

**Figure 1:**
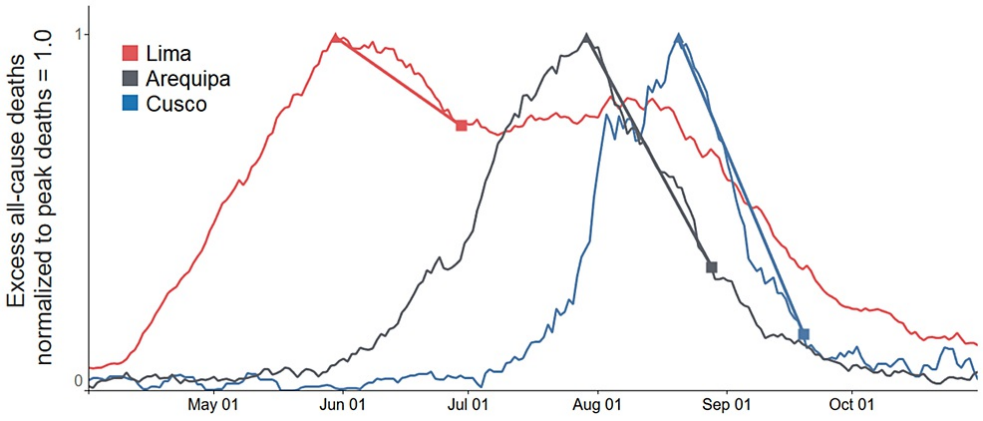
Excess deaths, normalized with peak deaths = 1.0 for each state One state is shown from each of the three tiers of IVM distribution: maximal, operation *MOT* (Cusco); moderate (Arequipa); and minimal (Lima). ▲ date of peak deaths; ■ 30 days after date of peak deaths. In Lima, after restrictions on IVM distribution through July 2020, distributions and treatments began on a mass basis in August [20,24,25]. Excess deaths shown are seven-day moving averages for ages ≥60; data are from Peru’s National Death Information System (SINADEF) [13,76].

Excess all-cause deaths were calculated from total deaths, state by state, by subtracting the respective baseline means for January through February 2020. This simple normalization procedure was reasonable given the small variations in deaths per month in Peru from January 2017 through February 2020. During this period, monthly all-cause deaths fluctuated with a mean value of 5.2% and a standard deviation of 3.8% [14]. However, total deaths in Peru beginning in May 2020 fluctuated by more than double the baseline value in January through February 2020, reflecting the impact of the pandemic [14]. For each of Peru’s 25 states, in individuals aged 60 and above, the date of peak (all-cause) deaths was determined to be the date after March 1, 2020, when the seven-day moving average of deaths reached maximum value in that state’s first wave of rising deaths from the pandemic. Excess deaths were then also tracked at 30 and 45 days following the date of peak deaths. Figure 1 provides examples of graphs of seven-day moving averages of excess deaths for three states, with line segments joining values of excess deaths at the date of peak deaths and 30 days following.

Determination and de-association of extraneous influences on excess deaths

To minimize potential distortions caused by varying proportions of younger or older people in the population of any given state of Peru or by potential changes in the percentage of COVID-19 cases across different age groups, all analyses except that for the changes in nationwide excess deaths were restricted to the population age ≥60, as obtained by filtering the database records by age. As noted earlier, because 75% of the excess deaths linked to COVID-19 during the pandemic’s first wave in Peru in 2020 occurred in the subpopulation of age ≥60 [18], this selection did not significantly change the analysis, but it eliminated one potential confounding factor. As noted above, only excess deaths, not COVID-19 case incidence or case fatalities, were used to track mortality associated with the pandemic in Peru due to the unreliability of the last two values.

The potential effects of Peruvian policies to limit social interactions were also considered. Peru implemented a two-week national lockdown on May 16, 2020, extended through the end of June, which ordered the closing of national borders and restriction of domestic travel and all non-essential activities [2]. However, as a Latin American policy official summarized, this lockdown “failed completely” because for 75% of Peruvian residents, “if they do not work one day, they cannot eat” [2]. An indication of actual compliance to such official lockdown orders is provided by Google Community Mobility Report data from cell phones within a given locality, which allow an objective quantification of social interactions [78-81]. Actual versus mandated changes in social mobility elsewhere in the world were found to have likewise varied considerably during the 2020 pandemic period. In some countries, such as Sweden, certain mobility restrictions were undertaken on individual initiatives [79], while in others, official mandates had limited impact on actual mobility [80,81]. Therefore, to most objectively assess the potential effects of social isolation policies on mortality trends in Peru, six indices of the Google Community Mobility Report data were retrieved for each of Peru’s 25 states and compared with mortality trends.

## Results

**Figure 2:**
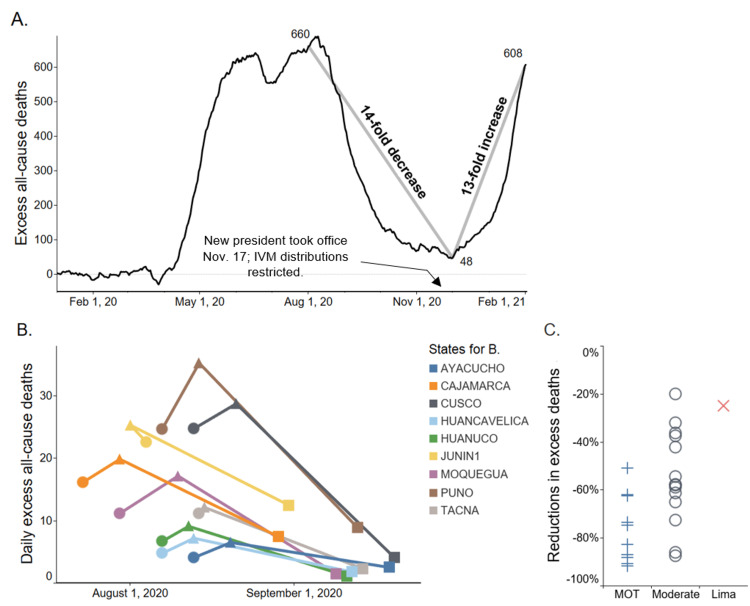
Excess all-cause deaths, for the national population of Peru and by state, overview A) Excess all-cause deaths (all ages), national population of Peru. For A and B, the *y* values are seven-day moving averages; for B and C, ages ≥60. Data are from Peru’s National Information System of Deaths (SINADEF) [76], with associated frozen datasets available from the Dryad data repository [13]. B) Reductions in excess deaths for all states of *Mega-Operación Tayta* (MOT), a program of mass ivermectin (IVM) distributions, excluding Pasco, which had them on three different dates. ● *MOT* start date; ▲ date of peak deaths; ■ 30 days after date of peak deaths. (Junin had a distribution of IVM 13 days before the *MOT* start.) C) Reductions in excess deaths at 30 days after peak deaths for the 25 states, by extent of IVM distributions: maximal-*MOT* (+), mean ‑74%; moderate-local distributions (◯), mean -53%; and minimal-Lima (x), -25%.

Analysis on excess all-cause deaths was performed state by state, with the population ages ≥60. For each state, the date of peak excess deaths in its first wave of the pandemic, as specified in the Materials and Methods section, was determined. Decreases in excess deaths from the date of peak deaths to 30 and 45 days afterwards were then tracked. The 25 states of Peru were grouped by the extent of IVM distributions: maximal (mass IVM distributions through operation *MOT*, 10 states), medium (locally managed IVM distributions, 14 states), and minimal (Lima, with restrictive policies). Table 1 shows the mean reductions in excess deaths at 30 and 45 days after date of peak deaths by the tier of IVM distribution, as also shown graphically for +30 days after peak deaths in Figure 2C. Table 2 shows these values for each state individually.

**Table 1:** Reductions in excess deaths at 30 and 45 days after date of peak deaths Excess all-cause deaths are seven-day moving averages, ages ≥60. These are shown grouped by the maximal, medium, or minimal extent of IVM distributions. The Kendall tau and Spearman rho and their associated p-values are for the absolute value of reduction in excess deaths at +30 days and +45 days after peak deaths correlated with tier of IVM distributions, by state. IVM: ivermectin; MOT: Mega-Operación Tayta

State	Peak excess deaths	+30 days		+45 days	
		Value	Change	Value	Change
Maximal IVM distributions through operation *MOT* (10 states)	164.9	42.3	-74.4%	22.7	-86.2%
Medium scale, locally managed IVM distributions (14 states)	396.8	187.3	-52.8%	120.3	-69.7%
Minimum scale, restricted IVM distributions (Lima)	263.6	197.6	-25.0%	197.2	-25.2%
All 25 states, Total	825.3	427.2	-48.2%	340.2	-58.8%
Kendall tau			τ_b_=0.5238, p=0.0019		τ_b_=0.4869, p=0.0039
Spearman rho			ρ=0.6188, p=0.0010		ρ=0.5764, p=0.0026

**Table 2:** Seven-day moving average of excess deaths, by state Seven-day moving averages are for ages ≥ 60, 30 and 45 days after the dates of peak deaths. Data are from Peru National Information System of Deaths (SINADEF) database [13,76].

State	Population age ≥ 60	Peak excess deaths		deaths +30 days		Deaths +45 days	
		Date	Value	Value	Change	Value	Change
Amazonas	35,174	Jul 25	4.4	0.7	-84.1%	0.9	-79.5%
Ancash	150,716	Jun 15	22.0	13.2	-40.0%	14.6	-33.6%
Apurimac	41,253	Sep 23	5.4	2.3	-57.4%	1.6	-70.4%
Arequipa	212,228	Jul 28	64.3	22.5	-65.0%	9.5	-85.2%
Ayacucho	62,206	Aug 20	6.5	2.5	-61.5%	1.5	-76.9%
Cajamarca	133,274	Jul 30	19.9	7.4	-62.8%	6.9	-65.3%
Callao	178,909	May 21	42.0	28.5	-32.1%	18.8	-55.2%
Cusco	138,969	Aug 21	28.8	4.0	-86.1%	0.0	-100.0%
Huancavelica	30,834	Aug 13	7.2	1.8	-75.0%	1.8	-75.0%
Huánuco	63,505	Aug 12	9.1	1.1	-87.9%	1.8	-80.2%
Ica	118,348	Jul 13	25.5	16.7	-34.5%	10.9	-57.3%
Junín	149,830	Aug 1	25.3	12.5	-50.6%	4.0	-84.2%
La Libertad	257,655	Jun 22	55.0	35.2	-36.0%	22.4	-59.3%
Lambayeque	177,031	May 15	30.4	13.8	-54.6%	8.5	-72.0%
Lima	1,648,028	May 30	263.6	197.6	-25.0%	197.2	-25.2%
Loreto	84,137	May 6	36.3	10.0	-72.5%	6.4	-82.4%
Madre De Dios	15,441	Jun 24	4.8	1.9	-60.4%	1.1	-77.1%
Moquegua	29,157	Aug 10	17.2	1.5	-91.3%	0.9	-94.8%
Pasco	26,384	Aug 7	3.5	0.3	-91.4%	0.5	-85.7%
Piura	234,250	May 24	58.5	24.9	-57.4%	15.7	-73.2%
Puno	144,017	Aug 14	35.3	8.9	-74.8%	4.7	-86.7%
San Martin	79,911	Jun 22	16.4	10.3	-37.2%	5.9	-64.0%
Tacna	49,376	Aug 15	12.1	2.3	-81.0%	0.6	-95.0%
Tumbes	28,166	Jun 2	9.7	4.3	-55.7%	2.0	-79.4%
Ucayali	51,639	May 12	22.1	3.0	-86.4%	2.0	-91.0%

Note that for Lima, as detailed in a previous analysis [20], large distributions of IVM beginning in August marked the end of its prior policy of IVM restrictions, with a second peak in excess deaths occurring on August 4, followed by a decline, as shown in Figure 1. However, as per the methodology of this analysis, the date of Lima’s first peak in excess deaths, May 30, was used, which occurred during the period in which its IVM distributions were minimal, per restrictions then in place.

As shown in Table 1, the mean reductions in excess deaths 30 days after date of peak deaths were 74%, 53%, and 25% for the maximal, medium, and minimal IVM distribution states, respectively. Figure 2C shows these drops in excess deaths over 30 days for the 25 states, by tier of IVM distribution. At 45 days after peak deaths, these mean reductions were 86%, 70%, and 25%, respectively. For nine of the 10 *MOT* states (excluding Pasco, which had three IVM distribution dates), *MOT* start dates were plotted together with dates of peak deaths in Figure 2B. As shown there, excess deaths dropped sharply in close time conjunction with the *MOT* start dates. Except for Junin, which had additional IVM distributions 13 days before its *MOT* start date, the lag time between the *MOT* start date and date of peak deaths varied from one to 11 days.

For analysis using the Kendall τb correlation, a statistical methodology appropriate for ordinal, categorical variables as used here, the three groups of states were assigned extent of IVM distribution values of 0 for Lima, 1 for the 14 local IVM distribution states, and 2 for the 10 *MOT* states. For correlations between the extent of IVM distributions and reductions in excess deaths (absolute values) at 30 days after peak deaths, a Kendall tau calculation [82] yields a τb value of 0.524, with an associated two-tailed p-value of 0.0019. However, because the hypothesis being tested is whether there was a positive correlation between the extent of IVM distribution and (absolute value of) reduction in excess deaths by state, the one-tailed p-value of 0.00096 is most appropriate, with the two-tailed p-value being an overly conservative measure of statistical significance. For the extent of IVM distribution correlated with reductions in excess deaths at 45 days after peak deaths, Kendall τb was 0.487, with a two-tailed p-value of 0.0039 and one-tailed p-value of 0.00195.

For the time periods of both 30 and 45 days after peak excess deaths, correlations between reductions in excess deaths and the extent of IVM distributions were considerably sharper than the threshold for an established clinical effect, as manifested by associated p-values much less than 0.05. These overall sharp correlations were obtained despite anomalies, such as high levels of public and private distributions of IVM in some non-*MOT* states, such as Loretto [20], which had a 73% drop in excess deaths at 30 days after peak. Meanwhile, Callao, which had a 32.1% reduction in excess deaths 30 days after peak deaths, the second lowest after that for Lima, is entirely contained within the state of Lima and may have had similar restrictions in IVM distributions through July 2020 as occurred in Lima.

**Figure 3:**
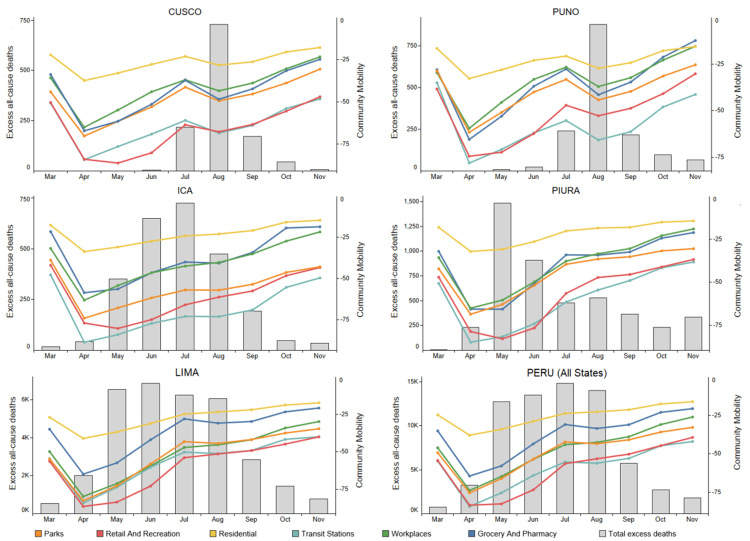
Google community mobility indices (line graphs) and excess all-cause deaths (bars) These mobility indices [83] show percentage changes in different categories relative to the median of these daily figures for January 3 through February 6, 2020. Five of these categories, all but residential, show (reduced) percentages of trips to various destinations with respect to this baseline, while the residential index tracks hours spent at home; for this category, the sign is switched so that, e.g., a 25% increased time at home appears as -25%. These graphs are for two* Mega-Operación Tayta (MOT)* states (Cusco and Puno), two states with local IVM distributions (Ica and Piura), Lima, and all of Peru. Excess all-cause deaths [13,76] are for ages ≥60 (bars). These same graphs have been generated for each of the 25 states of Peru [14].

As shown in Figure 3, concurrent with the reduction in COVID-19 excess deaths in each state after its month of peak deaths, there were increases in six Google-tracked indices of community mobility. These mobility indices show a similar pattern among states: a sharp decline from March to April 2020, followed by a steady rise through November, with a brief and modest decrease in August. There were no reductions in mobility that can explain the reductions in excess deaths shown in Figure 3 and as also shown for all 25 states [14].

Nationwide changes in daily excess all-cause deaths (seven-day moving average, all ages) before and after IVM use was restricted support the possibility that IVM treatments for COVID-19 were effective in Peru in that timeframe. As shown in Figure 2A, between August 1 and December 1, 2020, during the period of IVM use, nationwide excess deaths decreased by 14-fold. After IVM use was restricted [13,75], however, excess deaths then increased 13-fold through February 1, 2021.

## Discussion

The 25 states of Peru that conducted IVM treatments for COVID-19 at different time periods provide a robust set of subpopulations in which the treatment impacts can be evaluated. For the 10 *MOT* states, excess deaths dropped most sharply, by mean values of 74% at +30 days and 86% at +45 days after the date of peak deaths. For the 14 states with locally administered IVM distributions, excess deaths dropped by means of 53% at +30 days and 60% at +45 days. In Lima, however, where IVM treatments were delayed until August, four months after its initial pandemic surge in April, excess deaths dropped by only 25% at +30 days and by only 25% at +45 days after its date of peak deaths on May 30. The *MOT* states had sharp drops in excess deaths after reaching peak values in close time conjunction after *MOT* start dates (Figure 2B). For the full set of 25 states, reductions in excess deaths correlated with the extent of IVM distribution, maximal, medium, or minimal, with p<0.002 using the (two-tailed) Kendall τb test.

Given the association between IVM treatments and sharp mortality reductions revealed in this analysis, neither random fluctuation nor an unidentified, extraneous cause of these reductions in deaths appears likely. However, it is useful to consider the potential confounding influences of social isolation, changing seropositivity rates, variations in viral strains across states, and other factors. To begin with the most straightforward of these considerations, potential distortions caused by varying proportions of younger or older people in the population of any given state of Peru or by potential changes in percentage of COVID-19 cases across different age groups were ruled out by including only the population age 60 and above in the analysis. Moreover, for each of the 25 states of Peru, for the subpopulation of age ≥60, it was found that no more than 2.2% of that group died during the period of March-November 2020 [14]. Reductions of at most 2.2% of the total population aged ≥60 in each state were very small in comparison to pandemic-related fluctuations of more than 200% in all-cause deaths in 2020 [14].

The possibility that a more virulent strain of SARS-CoV-2 caused more fatalities in Lima than elsewhere in Peru was discounted by an analysis of 149 genomes from COVID-19 patients in Peru obtained through July 4, 2020 from diverse geographical regions of the country [16]. This genomic analysis found that the phylogenetic clades in 11 states had a distribution similar to that of Lima. Note that the UK variant of SARS-CoV-2, first detected in Peru on January 8, 2021 [84], cannot explain the post-November 2020 national surge in excess deaths, as shown in Figure 2A, because prior to then, excess deaths (all ages) had already tripled from 48 on December 1 to 150 on January 1 [13,76]. A Pan-American survey found that no other mutations of potential interest to public health, including 501Y.V2 and P.1, were detected in Peru as of mid-January 2021 [85].

The possibility that varying compliance with social isolation mandates in the different states of Peru could account for varying impacts of the pandemic is discounted by Google community mobility data shown in Figure 3. These data demonstrate that for Lima, the 10 *MOT* states and the 14 states with local IVM distributions, mobility patterns from March through November 2020 were roughly the same and that excess deaths fell in all states except Lima as mobility rose in their respective first waves of the pandemic.

The possibility that developing herd immunity was responsible for the major reductions in excess deaths seen in almost every state of Peru but Lima is discounted by the consideration of state-by-state seropositivity rates for November 2020 [14]. Although a high seropositivity rate for Loreto, which had reached 75% even by September [86], could explain reduced pandemic impacts there, several other IVM-treated states with low seropositivity rates had sharp drops in COVID-19 mortality. For Cajamarca, Cusco, Huancavelica, and Tacna, for example, all *MOT* states, seropositivity rates were only 20%, 18%, 18%, and 15%, respectively, in November 2020. However, within one to eight days after the *MOT* start date, excess deaths peaked and then dropped over 30 days, by 63%, 86%, 75%, and 81%, respectively. For the state-by-state correlation of reduction in excess deaths at date of peak deaths plus 30 days with seropositivity rate for November 2020, the Pearson’s p-value was 0.486, while the correlation for reduction in excess deaths at +45 days had a p-value of 0.415, showing no association and discounting any such dependence.

To consider the potential confounding influence of population density, even though Lima has the highest population density per area in Peru, with 10,577 inhabitants per km^2^ [87], densities for other cities were not much lower. Inhabitants per km^2^ in Trujillo, the capital of La Libertad, was 9,431; this figure was 8,216 for Piura and 8,195 for Cusco [87]. As for people living in the same household, a demographic study in 2017 showed that Lima households with more than five people represented 27% of the total; in Loreto, that figure was 42%, and in Ucayali, 36% [14,88]. Thus, neither population densities per area nor densities per household were markedly different in Lima versus population centers of other states for which this analysis was performed.

It had been proposed that cross-immunity from the dengue virus, which causes dengue fever, could explain lower than expected levels of COVID-19 mortality in some regions of South America, including Brazil [89]. This theory collapses in Peru, however, with greater than 70% reductions at 30 days after peak deaths, for example, in both the Peruvian states of Moquegua, which has not had dengue cases in the last 20 years, and Loreto, the epicenter of dengue in Peru [90,91]. Finally, one other data artifact could be that several peaks and drops in Lima's different districts could explain the low reduction in excess deaths. However, the pattern of total deaths, ages ≥60, for most of these districts, those comprising the bulk of the population, is the same: rising deaths to a peak around late May 2020 and then a three-month plateau following [14].

One potential confounding factor that cannot be conclusively resolved is the potential role in the operation *MOT* of the distribution of IVM for prophylaxis to members of the households of COVID-19 patients and others at high risk for the disease who had not tested positive for it (see section on *MOT* above). A preventative effect of IVM against COVID-19 may have contributed to the sharp reductions in excess deaths that followed its use in the 10 *MOT* states. The potential efficacy of IVM for COVID-19 prevention was indicated in two human prophylaxis studies, both using IVM in doses of at least 150 μg/kg per week. These RCTs reported statistically significant (p<0.001) reductions of 87% and 83% in COVID-19 incidences as compared with controls, with 93% and 100% reductions, respectively, in incidences of severe cases [92,93]. A third IVM prevention RCT reported a 72% reduction in COVID-19 incidence (p<0.0001) [94]. A fourth RCT for COVID-19 prevention administered just one dose of IVM at 12 mg (about 150 μg/kg) to 617 subjects on day one of a 42-day observation period, while three other agents were each administered daily to other study arms over that same period [95]. IVM at that single low dose on day one yielded the best results of the four agents, with highly statistically significant reductions of close to 50% in both symptomatic COVID-19 (p=0.01) and acute respiratory symptoms (p=0.0034) versus controls. A prospective observational study of 54,000 subjects in Itajaí, Brazil, found an 86% reduction in COVID-19 mortality in regular IVM users versus controls [96].

Thus, the prophylactic use of IVM may have contributed to the mean 74% reduction in excess deaths at 30 days after peak deaths in the 10 *MOT* states, and varying degrees of prophylactic use in the 14 non-*MOT* states that distributed IVM may have contributed to the 53% mean reduction in excess deaths over that period in those states. A preventative effect by IVM against COVID-19 may have likewise contributed to the 14-fold reduction in excess deaths nationwide from August 1 through December 1, 2020, as charted in Figure 2A. Given IVM’s proven record of safety in 3.7 billion human doses worldwide since 1987 (see section on the background on IVM treatments of COVID-19), the use of preventative dosing to complement the administration of treatments would be reasonable to consider for any national deployment against COVID-19. Its low cost is another factor that makes mass distribution feasible. The possibility of both preventative and treatment efficacies of IVM was raised by outcomes in another world region in which IVM was distributed to the population at risk for COVID-19 on a mass scale. This IVM distribution occurred in Uttar Pradesh, the largest state in India, having a population of 229 million. As the World Health Organization reported on May 7, 2021, government teams moved across 97,941 villages in 75 districts in a COVID-19 management program that began two days earlier on May 5 [97]. As Uttar Pradesh government sources describe, central to this COVID-19 mitigation program was the distribution of home medication kits consisting of IVM, doxycycline, zinc, vitamins C and D3, and paracetamol (acetaminophen) tablets [98,99].

On May 7, 2021, the date of this WHO report, the seven-day moving average of COVID-19 deaths per million of population was roughly the same in Uttar Pradesh, all of India, and the United States, i.e., 1.4, 3.2, and 1.9, respectively [100]. Between May 7, 2021 and July 7, 2021, however, after the beginning of the mass distribution of IVM, the seven-day moving average of COVID-19 deaths in Uttar Pradesh fell by 97%, from 328 to 10. The cumulative total of COVID-19 deaths per million in population from July 7, 2021 through April 1, 2023 was 4.3 in Uttar Pradesh, as compared with 70.4 in all of India and 1,596.3 in the United States [100]. These data are from the Institute for Health Metrics and Evaluation (IHME), University of Washington (Seattle, USA), as had been reported through December 2022 and modeled thereafter through April 1, 2023. The much lower number of COVID-19 deaths per population in all of India versus the United States in that period may reflect the use of these same home treatment kits containing IVM, doxycycline, and zinc in some other states of India [99,101]. The extent of COVID vaccine coverage of these three populations between May 7, 2021 and April 1, 2023, lowest in Uttar Pradesh and highest in the United States [100], cannot explain the trends in COVID-19 deaths that occurred.

Unlike for Peru, where granular data by state and by time period for excess deaths allow a well-founded correlation of trends for excess deaths with the extent of IVM use, the data for Uttar Pradesh are suggestive but not conclusive for efficacy of IVM in the treatment and possibly also the prevention of COVID-19. The possibility of such efficacy having been achieved in Uttar Pradesh, however, is supported by the particularly high efficacy of IVM in conjunction with doxycycline and zinc in COVID-19 treatment (the combination used in Uttar Pradesh) as reported in two clinical studies [66,67].

The exceptional safety profile and low cost of IVM certainly support its use as in Peru’s operation *MOT* and in Uttar Pradesh as an attractive national policy for COVID-19 mitigation. These significant reductions in mortality as achieved in Peru and Uttar Pradesh suggest that the impact of such a national IVM deployment would be observable within a relatively short period. However, generic drugs have often fared poorly in competition with patented offerings in past decades, based upon the unfortunate vulnerability of science to commodification and regulatory capture [102,103]. For example, the inexpensive triple therapy for peptic ulcers, proven 96% curative in 1990 and now the standard of care, was delayed for a decade in its worldwide deployment until patents for two best-selling palliative drugs for peptic ulcers expired [48]. Such a potential bias against IVM was suggested by a February 4, 2021 press release from Merck, which was then developing its own patented COVID-19 therapeutic, claiming that there was “a concerning lack of safety data” for IVM [104]. However, IVM is Merck’s own drug, found safe at doses considerably higher than its standard dose in several studies, as cited in the section on the background on IVM treatments of COVID-19, and the Nobel Prize committee specifically noted IVM’s safety record in honoring the discovery of this drug in its 2015 prize for medicine [105].

## Conclusions

The natural experiment that was put into motion with the authorization of IVM use for COVID-19 in Peru on May 8, 2020, resulted in strong evidence for the drug's effectiveness. Across the 25 states of Peru, there was a significant correlation between the extent of IVM distributions and reductions in excess deaths over 30 days (p<0.002). The 10 states involved in operation *MOT*, having the greatest extent of IVM distributions, saw a mean reduction in excess deaths of 74% at 30 days after peak deaths. Several potential confounding factors, including effects of a social isolation mandate imposed in May 2020, variations in the genetic makeup of the SARS-CoV-2 virus, and differences in seropositivity rates and population densities across the 25 states, were considered but did not appear to have significantly influenced these outcomes.

These encouraging results from IVM treatments in Peru and similar positive indications from Uttar Pradesh, India, which have populations of 33 million and 229 million, respectively, offer promising models for further mass deployments of IVM, as needs may arise, for both the treatment and prevention of COVID-19. IVM's proposed mechanism of action against SARS-CoV-2, which entails drug binding to the virus' spike protein, limiting the virus’ morbidity and infectivity, may allow the efficacy of IVM to be conserved against viral mutant strains that may emerge. Further investigations will help to establish the generalizability of these findings, for example, to therapeutic applications to post-acute sequelae of COVID-19 (PASC), and to explore the potential use of IVM for the treatment and prevention of COVID-19 as the pandemic continues to evolve.

## References

[1] (2023). First case of coronavirus in Peru: the history of contagion in the pilot [Article in Spanish]. https://gestion.pe/peru/primer-caso-de-coronavirus-en-peru-los-detalles-del-contagio-del-piloto-noticia/.

[2] Welsh T (2023). Inequality and corruption: why Peru is losing its COVID-19 battle. https://www.devex.com/news/inequality-and-corruption-why-peru-is-losing-its-covid-19-battle-97604.

[3] Ministry of Health (May 8, 2020). Ministerial Resolution #270-2020 [Article in Spanish].

[4] Yagisawa M, Foster PJ, Hanaki H, Omura S (2021). Global trends in clinical studies of ivermectin in COVID-19. Jpn J Antibiot.

[5] Juarez M, Schcolnik-Cabrera A, Dueñas-Gonzalez A (2018). The multitargeted drug ivermectin: from an antiparasitic agent to a repositioned cancer drug. Am J Cancer Res.

[6] Campbell WC (2012). History of avermectin and ivermectin, with notes on the history of other macrocyclic lactone antiparasitic agents. Curr Pharm Biotechnol.

[7] Turkewitz J, Kurmanaev A (2023). New York Times: Peru chooses 3rd president in a week amid street protests. New York Times. November 16.

[8] (2023). Mazzetti on ivermectin: Minsa cannot freely deliver drugs without international accreditation [Article in Spanish]. https://elcomercio.pe/lima/sucesos/pilar-mazzetti-sobre-ivermectina-minsa-no-puede-entregar-farmacos-sin-acreditacion-internacional-coronavirus-peru-covid-19-nndc-noticia/.

[9] Redacción RPP (2023). Violeta Bermúdez: "If a doctor evaluates a person and prescribes ivermectin, they can use it" [Article in Spanish]. RPP. January 22.

[10] (2023). COVID-19: Vizcarra recommended the use of ivermectin to treat disease [Article in Spanish]. https://canaln.pe/actualidad/covid-19-martin-vizcarra-recomendo-uso-ivermectina-tratamiento-enfermedad-n429808.

[11] Gorriti G. El Rompeolas. IDL Reporteros (2023). Breakwater. IDL Reporters [Article in Spanish]. January 20.

[12] Gorriti G (2023). From the scarf to ivermectin [Article in Spanish]. IDL Reporteros. February 3.

[13] (2023). Dryad data repository; frozen data snapshots from the Peruvian national SINADEF death information system from December 13, 2020 and February 23, 2021 and associated data documentation. Dryad Data Repository. (2021). https://datadryad.org/stash/dataset/doi:10.5061/dryad.dv41ns1xr.

[14] Chamie-Quintero JJ, Hibberd JA, Scheim D (2023). Death seasonality, Google community mobility trends, seropositivity rates, comparisons of SINADEF data with who summary data, and other data items as useful in analysis of excess deaths during the COVID-19 pandemic in Peru, 2020-2021 (Chamie-Quintero JJ, Hibberd, JA, Scheim DE) [PREPRINT]. OSF Preprints.

[15] (2023). Covid-19: second report to update the death toll will be released this week. Andina Peruvian News Agency [Article in Spanish]. https://andina.pe/agencia/noticia-covid19-segundo-informe-para-actualizar-cifra-fallecidos-se-conocera-esta-semana-807333.aspx.

[16] Juscamayta-López E, Carhuaricra D, Tarazona D, Valdivia F, Rojas N, Maturrano L, Gavilán R (2021). Phylogenomics reveals multiple introductions and early spread of SARS-CoV-2 into Peru. J Med Virol.

[17] Padilla-Rojas C, Vega-Chozo K, Galarza-Perez M (2020). Genomic analysis reveals local transmission of SARS-CoV-2 in early pandemic phase in Peru. bioRxiv.

[18] Sempé L, Lloyd-Sherlock P, Martínez R, Ebrahim S, McKee M, Acosta E (2021). Estimation of all-cause excess mortality by age-specific mortality patterns for countries with incomplete vital statistics: a population-based study of the case of Peru during the first wave of the COVID-19 pandemic. Lancet Reg Health Am.

[19] Republic of Peru (2016). Political Constitution of Peru Edition of the Congress of the Republic [Article in Spanish].

[20] Chamie-Quintero JJ, Hibberd JA, Scheim DE (2022). Sharp reductions in COVID-19 case fatalities and excess deaths in Peru in close time conjunction, state-by-state, with ivermectin treatments (Chamie-Quintero JJ, Hibberd, JA, Scheim DE). OSF Preprints.

[21] (2023). Ivermectina: crece la demanda de fármaco antiparasitario para casos de covid-19. Realidadpe | Noticias relevantes del Perú | Francisco Sagasti, Elecciones 2021, COVID-19, Reactiva Perú, Arranca Perú, Cuarentena focalizada. April 29, 2020. https://realidad.pe/salud/ivermectina-crece-la-demanda-de-farmaco-antiparasitario-para-casos-de-covid-19/.

[22] (2023). Piura: a couple is intervened with more than 200 doses of contraband ivermectin [Article in Spanish]. https://elcomercio.pe/peru/piura-detienen-a-dos-personas-con-mas-de-200-dosis-de-ivermectina-de-contrabando-coronavirus-covid-19-nnpp-noticia.

[23] (2023). San Martín de Porres: 20,000 vials of ivermectin seized and 12 people arrested [Article in Spanish]. Andina Agencia Peruana de Noticias.

[24] El Comercio (2023). El Comercio: A trip to the black market of COVID-19 [Article in Spanish]. https://www.facebook.com/watch/?v=292349115157434.

[25] (2023). COVID-19 in Peru: Pharmacy in SJM that sold adulterated ivermectin and drugs stolen from the State intervened [Article in Spanish]. https://elcomercio.pe/lima/sucesos/intervienen-farmacia-en-sjm-que-vendia-invermectina-adulterada-y-medicamentos-sustraidos-del-estado-noticia/?ref=ecr.

[26] Datos Abiertos de COVID-19; Plataforma Nacional de Datos Abiertos (2023). COVID-19 Open Data; National Open Data Platform. Digital Government Secretariat Presidency of the Council of Ministers [Article in Spanish]. https://www.datosabiertos.gob.pe/group/datos-abiertos-de-covid-19.

[27] (2023). More than 500 older adults are cared for by the Mimp during Operation Tayta in Pasco [Article in Spanish]. https://andina.pe/agencia/noticia-mas-500-adultos-mayores-son-atendidos-por-mimp-durante-operacion-tayta-pasco-807916.aspx.

[28] (2023). Management Resolution, No. 133-2020-HMPP-A/GM. Municipal, Provincial Management of Pasco [Article in Spanish]. https://www.munipasco.gob.pe/admin/files/1669x9xf7f741e17z7417z7ze17a79xf79xf78179x9xa73670E4g3670E4g3670E4g0E4g.pdf.

[29] (2023). Management Resolution, No. 154-2020-HMPP-A/GM. Municipal, Provincial Management of Pasco [Article in Spanish]. https://cdn.www.gob.pe/uploads/document/file/2065537/Resoluci%C3%B3n%20Gerencial%20%20N%C2%B0%20133%20-%20Aprobar%20el%20Plan%20de%20Trabajo%20denominado%20%22Operaci%C3%B3n%20TAYTA%20para%20Proteger%20a%20la%20Poblaci%C3%B3n%20Vulnerable%20ante%20el%20COVID-19%20en%20Zonas%20de%20Riesgo%22..pdf.

[30] (2023). Junín: regional government produces ivermectin for early treatment of COVID-19 [Article in Spanish]. Andina, Agencia Peruana de Noticias.

[31] (2023). Hospital Unanue maintains regular production of ivermectin. Regional Government of Tacna [Article in Spanish]. https://www.gob.pe/institucion/regiontacna/noticias/548716-hospital-unanue-mantiene-produccion-regular-de-ivermectina.

[32] (2023). Government starts Operation TAYTA to protect the vulnerable population against COVID-19 in risk areas. Lima, Peru: Ministry of Defense of Peru [Article in Spanish]. Lima, Peru: Ministerio de Defensa de.

[33] (2023). Plan for the actions of the multisectoral working group called "I take care of you Peru," Ministry of Defense, General Directorate of Policy and Strategy [Article in Spanish]. https://drive.google.com/file/d/1s-EQwuT59Na8umvxqWthXCo2Borl0JPG/view?usp=sharing.

[34] de Althaus, J J (2023). Mayor of Lima must convene business and government to apply massive family fences [Article in Spanish]. Lampidia.

[35] Yumbato A (2023). Coronavirus in Peru: find out how Operation Tayta works. https://rpp.pe/peru/actualidad/coronavirus-en-peru-conoce-como-funciona-la-operacion-tayta-noticia-1286058.

[36] (2023). Operation Tayta arrived in Huánuco to strengthen the fight against COVID-19 [Article in Spanish]. https://www.gob.pe/institucion/regionhuanuco/noticias/288607-operacion-tayta-llego-a-huanuco-para-fortalecer-lucha-contra-covid-19.

[37] (2023). Covid-19: more than 22,000 people were attended by Operation Tayta-Yo Me Apunto in Cajamarca. Andina, Peruvian News Agency [Article in Spanish]. https://andina.pe/agencia/noticia-covid19-a-mas-22000-personas-atendio-operacion-taytayo-me-apunto-cajamarca-808221.aspx.

[38] (2023). Moquegua: another 45 doctors arrive from Lima to reinforce the fight against the coronavirus. Editorial The Republic [Article in Spanish]. https://larepublica.pe/sociedad/2020/08/05/moquegua-llegan-otros-45-medicos-desde-lima-para-reforzar-lucha-contra-el-coronavirus-ivermectina-lrsd/.

[39] (2023). A team from the Ministry of Health carries out Operation Tayta and provides technical assistance in Moquegua. Ministry of Health [Article in Spanish]. https://www.gob.pe/institucion/minsa/noticias/286591-equipo-del-ministerio-de-salud-realiza-operacion-tayta-y-brinda-asistencia-tecnica-en-moquegua.

[40] Llantoy C. (2023). Through enrollment executed by 60 brigadistas of the mph and “protection”. Peru Provincial Municipality of Huancayo [Article in Spanish]. Municipalidad Provincial de Huancayo.

[41] (2023). Minister of Defense: Operation Tayta allows the strengthening of primary care for COVID-19 cases [Article in Spanish]. Ministro de Defensa.

[42] (2023). Puno: Starting tomorrow they will implement the Tayta operation led by the Peruvian Army [Article in Spanish]. Radio Onda Azul.

[43] (2023). Operation Tayta will serve 10,000 vulnerable people in Huánuco. Andina, Peruvian News Agency [Article in Spanish]. https://andina.pe/agencia/noticia-operacion-tayta-atendera-a-10000-personas-vulnerables-huanuco-810025.aspx.

[44] Gobierno entrega a Huancavelica más de 17 000 unidades de EPP (2023). Government delivers to Huancavelica more than 17,000 units of PPE and medicines to fight Covid-19 [Article in Spanish]. https://www.gob.pe/institucion/produce/noticias/296401-gobierno-entrega-a-huancavelica-mas-de-17-000-unidades-de-epp-y-medicamentos-para-luchar-contra-el-covid-19.

[45] (2023). The Mega Tayta 2020 plan begins in ayacucho to diagnose, treat, isolate and assist positive cases with food [Article in Spanish]. https://www.gob.pe/institucion/regionayacucho/noticias/294735-inician-plan-mega-tayta-2020-en-ayacucho-para-diagnosticar-tratar-aislar-y-asistir-con-alimentos-a-los-casos-positivos.

[46] (2023). Covid-19: Tayta operation began with the application of 3,000 discard tests [Article in Spanish]. https://www.apnoticias.pe/peru/exitosa-noticias/covid-19-inicio-operacion-tayta-con-aplicacion-de-3000-pruebas-de-descarte-43904.

[47] Kory P, Meduri GU, Varon J, Iglesias J, Marik PE (2021). Review of the emerging evidence demonstrating the efficacy of ivermectin in the prophylaxis and treatment of COVID-19. Am J Ther.

[48] Santin AD, Scheim DE, McCullough PA, Yagisawa M, Borody TJ (2021). Ivermectin: a multifaceted drug of Nobel prize-honoured distinction with indicated efficacy against a new global scourge, COVID-19. New Microbes New Infect.

[49] López-Medina E, López P, Hurtado IC (2021). Effect of ivermectin on time to resolution of symptoms among adults with mild COVID-19: a randomized clinical trial. JAMA.

[50] Vallejos J, Zoni R, Bangher M (2021). Ivermectin to prevent hospitalizations in patients with COVID-19 (IVERCOR-COVID19) a randomized, double-blind, placebo-controlled trial. BMC Infect Dis.

[51] Reis G, Silva EA, Silva DC (2022). Effect of early treatment with ivermectin among patients with COVID-19. N Engl J Med.

[52] Lim SC, Hor CP, Tay KH (2022). Efficacy of ivermectin treatment on disease progression among adults with mild to moderate covid-19 and comorbidities: the I-TECH randomized clinical trial. JAMA Intern Med.

[53] Scheim Scheim, DE DE, Hibberd Hibberd, JA JA, Chamie-Quintero Chamie-Quintero, JJ. JJ. (2023). Protocol violations in López-Medina et al.: 38 switched ivermectin (IVM) and placebo doses, failure of blinding, ubiquitous IVM use OTC in Cali, and nearly identical AEs for the IVM and control groups (Scheim DE, Hibberd JA, Chamie-Quintero JJ) [PREPRINT]. OSF Preprints.

[54] Scheim Scheim, DE DE (2022). The drug used in Lim et al. 2022, source not specified, had <1% incidence of AEs distinctive and common for ivermectin at this study’s very high dose, 2 mg/kg (Scheim DE) [PREPRINT]. OSF Preprints.

[55] Scheim DE, Aldous C, Osimani B, Fordham EJ, Hoy WE (2023). When characteristics of clinical trials require per-protocol as well as intention-to-treat outcomes to draw reliable conclusions: three examples. J Clin Med.

[56] Table 4c (2023). NIH COVID-19 treatment guidelines. Fluvoxamine: selected clinical data, limitations and interpretation. Table 4c. https://www.covid19treatmentguidelines.nih.gov/tables/fluvoxamine-data/.

[57] (2023). Memorandum explaining basis for declining request for emergency use authorization of fluvoxamine maleate. U.S. Food & Drug Administration. https://www.accessdata.fda.gov/drugsatfda_docs/nda/2020/EUA%20110%20Fluvoxamine%20Decisional%20Memo_Redacted.pdf.

[58] Bramante CT, Huling JD, Tignanelli CJ (2022). Randomized trial of metformin, ivermectin, and fluvoxamine for Covid-19. N Engl J Med.

[59] Naggie S, Boulware DR, Lindsell CJ (2022). Effect of ivermectin vs placebo on time to sustained recovery in outpatients with mild to moderate COVID-19: a randomized clinical trial. JAMA.

[60] Naggie S, Boulware DR, Lindsell CJ (2023). Effect of higher-dose ivermectin for 6 days vs placebo on time to sustained recovery in outpatients with COVID-19: a randomized clinical trial. JAMA.

[61] Abdool Karim SS, Devnarain N (2022). Time to stop using ineffective COVID-19 drugs. N Engl J Med.

[62] Shafiee A, Teymouri Athar MM, Kohandel Gargari O, Jafarabady K, Siahvoshi S, Mozhgani SH (2022). Ivermectin under scrutiny: a systematic review and meta-analysis of efficacy and possible sources of controversies in COVID-19 patients. Virol J.

[63] Aminpour M, Cannariato M, Safaeeardebili ME (2022). In silico analysis of the multi-targeted mode of action of ivermectin and related compounds. Computation.

[64] Scheim DE (2022). A deadly embrace: hemagglutination mediated by SARS-COV-2 spike protein at its 22 n-glycosylation sites, red blood cell surface sialoglycoproteins, and antibody. Int J Mol Sci.

[65] Lehrer S, Rheinstein PH (2020). Ivermectin docks to the SARS-CoV-2 spike receptor-binding domain attached to ACE2. In Vivo.

[66] Stone JC, Ndarukwa P, Scheim DE (2022). Changes in SpO2 on room air for 34 severe COVID-19 patients after ivermectin-based combination treatment: 62% normalization within 24 hours. Biologics.

[67] Hazan S, Dave S, Gunaratne AW, Dolai S, Clancy RL, McCullough PA, Borody TJ (2022). Effectiveness of ivermectin-based multidrug therapy in severely hypoxic, ambulatory COVID-19 patients. Future Microbiol.

[68] Babalola OE, Ndanusa Y, Adesuyi A, Ogedengbe OJ, Thairu Y, Ogu O (2021). A randomized controlled trial of ivermectin monotherapy versus HCQ, IVM, and AZ combination therapy in Covid-19 patients in Nigeria. J Infect Dis Epidemiol.

[69] Crump A, Ōmura S (2011). Ivermectin, 'wonder drug' from Japan: the human use perspective. Proc Jpn Acad Ser B Phys Biol Sci.

[70] Chaccour C, Lines J, Whitty CJ (2010). Effect of ivermectin on Anopheles gambiae mosquitoes fed on humans: the potential of oral insecticides in malaria control. J Infect Dis.

[71] Guzzo CA, Furtek CI, Porras AG (2002). Safety, tolerability, and pharmacokinetics of escalating high doses of ivermectin in healthy adult subjects. J Clin Pharmacol.

[72] Navarro M, Camprubí D, Requena-Méndez A (2020). Safety of high-dose ivermectin: a systematic review and meta-analysis. J Antimicrob Chemother.

[73] de Castro CG Jr, Gregianin LJ, Burger JA (2020). Continuous high-dose ivermectin appears to be safe in patients with acute myelogenous leukemia and could inform clinical repurposing for COVID-19 infection. Leuk Lymphoma.

[74] Krolewiecki A, Lifschitz A, Moragas M (2021). Antiviral effect of high-dose ivermectin in adults with COVID-19: A proof-of-concept randomized trial. EClinicalMedicine.

[75] Buonfrate D, Chesini F, Martini D (2022). High-dose ivermectin for early treatment of COVID-19 (COVER study): a randomised, double-blind, multicentre, phase II, dose-finding, proof-of-concept clinical trial. Int J Antimicrob Agents.

[76] Ministerio de Salud de Peru (2021). National Death Information System (SINADEF); this dataset, updated daily, was accessible by the public through December 2022. Frozen data snapshots as used here are available in the Dryad data repository at this URL. https://datadryad.org/stash/dataset/doi:10.5061/dryad.dv41ns1xr.

[77] (2022). Estimated population by simple ages and age groups, according to department, province and district. Information Management Office, Ministry of Health, National Institute of Statistics and Informatics (INEI). National Censuses [Article in Spanish]. Censos Nacionales.

[78] Badr HS, Du H, Marshall M, Dong E, Squire MM, Gardner LM (2020). Association between mobility patterns and COVID-19 transmission in the USA: a mathematical modelling study. Lancet Infect Dis.

[79] Stokes J, Turner AJ, Anselmi L, Morciano M, Hone T (2022). The relative effects of non-pharmaceutical interventions on wave one Covid-19 mortality: natural experiment in 130 countries. BMC Public Health.

[80] Oh J, Lee HY, Khuong QL (2021). Mobility restrictions were associated with reductions in COVID-19 incidence early in the pandemic: evidence from a real-time evaluation in 34 countries. Sci Rep.

[81] Unwin HJ, Mishra S, Bradley VC (2020). State-level tracking of COVID-19 in the United States. Nat Commun.

[82] (2023). Wessa.net Free Statistics Software, Office for Research Development and Education, version 1.2.1. https://www.wessa.net/.

[83] (2020). Google COVID-19 Community Mobility Reports. https://www.google.com/covid19/mobility/.

[84] Cervantes M (January 9, 2020). Peru confirms case of British variant of coronavirus. Reuters.

[85] (2023). Epidemiological update: occurrence of variants of SARS-CoV-2 in the Americas - 26 January 2021. https://www.paho.org/en/documents/epidemiological-update-occurrence-variants-sars-cov-2-americas-26-january-2021.

[86] (2023). Covid-19: seroprevalence in the city of Iquitos is 75 percent. Andina Peruvian News Agency [Article in Spanish]. https://andina.pe/agencia/noticia-covid19-seroprevalencia-la-ciudad-iquitos-es-75-ciento-815197.aspx.

[87] (2023). European Comission, Global Human Settlement, Urban centres database 2018 visualisation; Urban Centre Database UCDB R2019A. https://ghsl.jrc.ec.europa.eu/ucdb2018visual.php.

[88] (2023). National Institute of Statistics and Informatics (INEI), National Censuses [Article in Spanish]. https://www.inei.gob.pe/media/MenuRecursivo/publicaciones_digitales/Est/Lib1539/cap06.pdf.

[89] Nicolelis MA, Raimundo RL, Peixoto PS, Andreazzi CS (2021). The impact of super-spreader cities, highways, and intensive care availability in the early stages of the COVID-19 epidemic in Brazil. Sci Rep.

[90] (2023). Ministry of Health, National Center for Epidemiology, Disease Prevention and Control, Health Situation Room. Ministry of Health, National Epidemiology Center [Article in Spanish]. https://www.dge.gob.pe/portal/docs/vigilancia/sala/2020/SE13/dengue.pdf.

[91] Cabezas C, Fiestas V, García-Mendoza M, Palomino M, Mamani E, Donaires F (2015). Dengue in Peru: a quarter of a century after its reemergence [Article in Spanish]. Revista Peruana de Medicina Experimental y Salud Publica.

[92] Shouman W, Hegazy A, Nafae R (2021). Use of ivermectin as a prophylactic option in asymptomatic family close contacts with patients of COVID-19 (NCT number: 04422561). J Clin Diagnostic Res.

[93] Chahla RE, Medina Ruiz L, Ortega ES (2021). Intensive treatment with ivermectin and iota-carrageenan as pre-exposure prophylaxis for COVID-19 in health care workers from Tucuman, Argentina. Am J Ther.

[94] (2023). The SAIVE Trial, post-exposure use of ivermectin in COVID-19 prevention: efficacy and safety results. Desort-Henin V, Kostova A, Babiker EA, Caramel A, Malamut R. Poster presentation, European Congress of Clinical Microbiology and Infectious Diseases. Desort-Henin, V.; Kostova, A.; Babiker, E. A.; Caramel, A.; Malamut, R. Poster presentation, European Congress of Clinical Microbiology and Infectious Diseases, April 18.

[95] Seet RC, Quek AM, Ooi DS (2021). Positive impact of oral hydroxychloroquine and povidone-iodine throat spray for COVID-19 prophylaxis: An open-label randomized trial. Int J Infect Dis.

[96] Kerr L, Baldi F, Lobo R (2022). Regular use of ivermectin as prophylaxis for COVID-19 led up to a 92% reduction in COVID-19 mortality rate in a dose-response manner: results of a prospective observational study of a strictly controlled population of 88,012 subjects. Cureus.

[97] (2023). Uttar Pradesh going the last mile to stop COVID-19. World Health Organization. World Health Organization. 7 May.

[98] Boretti A (2022). Zinc augments the antiviral potential of HCQ/CQ and ivermectin to reduce the risks of more serious outcomes from COVID-19 infection. J Trace Elem Med Biol.

[99] (2023). The miracle not-heard around the world: the success of Uttar Pradesh - part 3. https://pierrekory.substack.com/p/the-miracle-not-heard-around-the-1ee.

[100] (2023). Spreadsheet and screenshots of COVID-19 deaths, 7-day averages for selected dates, in Uttar Pradesh, all of India, and the United States, with underlying data (https://covid19.healthdata.org/) from The Institute for Health Metrics and Evaluation (IHME) at the University of Washington (Seattle, USA). https://drive.google.com/file/d/1ww64SlaXI5KPgmzNf2aNu6pVxJvjVCVJ/view?usp=drive_link.

[101] Ray S. Indian State Will Offer Ivermectin To (2023). Indian state will offer ivermectin to entire adult population — even as WHO warns against its use as COVID-19 treatment. Forbes. May 11.

[102] Saltelli A, Dankel DJ, Di Fiore M, Holland N, Pigeon M (2022). Science, the endless frontier of regulatory capture. Futures.

[103] Ioannidis JP (2016). Evidence-based medicine has been hijacked: a report to David Sackett. J Clin Epidemiol.

[104] Scheim DE (2023). Merck’s deadly Vioxx playbook, redux: a debunked smear campaign against its competing drug—the FDA-approved, Nobel prize-honored ivermectin (Scheim DE). Trialsite News.

[105] Nobel Prize Committee (2023). The 2015 Nobel Prize in Physiology or Medicine, Press release, Solna, Sweden: The Nobel Assembly at Karolinska Institutet. https://www.nobelprize.org/prizes/medicine/2015/press-release/.

[106] (2023). Ivermectin for COVID-19 in Peru: 14-fold reduction in nationwide excess deaths, p<0.002 for effect by state, then 13-fold increase after ivermectin use restricted (Chamie-Quintero JJ, Hibberd JA, Scheim DE) [PREPRINT]. https://osf.io/9egh4/.

